# Calculus of the Size of a Grain of Wheat Detected by the New Ausculating Searcher

**Published:** 1878-09

**Authors:** Edmund Andrews

**Affiliations:** No. 6 Sixteenth Street, Chicago


### NOTES FROM PRIVATE PRACTICE.

### *Calculus of the Size of a Grain of Wheat Detected by the new Auscultating Searcher.*

In the June number of the JOURNAL AND EXAMINER, I described and figured a new searcher for detecting minute calculi or their fragments in the bladder. It will be remembered that the searcher was made hollow, and had attached to the external extremity a slender rubber tube and ear piece, by means of which the slightest touch of a bit of calculus in the bladder sent a distinct sound to the ear of the surgeon.

A recent case illustrates the extreme delicacy of this instrument. Mr. A. B. had, a few weeks ago, an attack of severe pain, which, from his description, I presume to have been caused by the descent of a small calculus along the ureter. From this time on, he had mild symptoms of stone in the bladder and came to the city for treatment. Introducing the auscultating searcher, I distinctly heard the tick of a stone, which was so small that it gave no jar perceptible to the fingers. I immediately introduced a very small lithotrite, seized the calculus, and drew it out. It was about as large as a grain of wheat. I searched repeatedly for other fragments during the next few days, but found no more, and no further symptoms of stone appearing, I dismissed the patient as cured. This case afforded a most satisfactory test of the delicacy of the instrument.

Since describing my instrument in the June number of this journal, Sir Henry Thompson has brought out quite a different one in London, having the same object in view. He has a galvanic battery and a microphone attached to an ordinary searcher, so as to magnify the sound produced by striking the stone. The apparatus is extremely ingenious, and gives the sounds well, but the formidable incumbrance of a battery, a microphone, etc., must seriously impair its convenience. On that account, I cannot help thinking that my instrument will be found the most useful.

EDMUND ANDREWS, M. D.
NO. 6 SIXTEENTH STREET, CHICAGO.

